# Flexible Transparent Films of Oriented Silver Nanowires for a Stretchable Strain Sensor

**DOI:** 10.3390/ma17164059

**Published:** 2024-08-15

**Authors:** Xiaoguang Wang, Chengjun Song, Yangyang Wang, Shaoxuan Feng, Dong Xu, Tingting Hao, Hongbo Xu

**Affiliations:** 1 China Electronic Technology Group Corp 49th Research Institute, Harbin 150001, China; 15846328044@163.com (X.W.); cetc49_chengjun@163.com (C.S.); wyy307@126.com (Y.W.); hai-83107@163.com (S.F.); xudong_1114@126.com (D.X.); 2 School of Chemistry and Chemical Engineering, Harbin Institute of Technology, Harbin 150001, China

**Keywords:** silver nanowire, stretchable strain sensor, polydimethylsiloxane, flexible smart windows

## Abstract

The potential applications of stretchable strain sensors in wearable electronics have garnered significant attention. However, developing susceptible stretchable strain sensors for practical applications still poses a considerable challenge. The present study introduces a stretchable strain sensor that utilizes silver nanowires (AgNWs) embedded into a polydimethylsiloxane (PDMS) substrate. The AgNWs have high flexibility and electrical conductivity. A stretchable AgNW/Pat-PDMS conductive film was prepared by arranging nanowires on the surface of PDMS using a simple rod coating method. Depending on the orientation angle, the overlap area between nanowires varies, resulting in different levels of separation under a given strain. Due to the separation of the nanowire and the change in current path geometry, the variation in strain resistance of the sensor can be primarily attributed to these factors. Therefore, precision in strain regulation can be adjusted by altering the angle θ (0°, 60°, or 90°) of the nanowire. At the same time, the stability of the AgNW/Pattern-PDMS (AgNW/Pat-PDMS) conductive film application was verified by preparing a sandwich structure PDMS/AgNW/Pat-PDMS stretchable strain sensor. The sensor exhibited high sensitivity within the operating sensing range (gauge factor (GF) of 15 within ~120% strain), superior durability (20,000 bending cycles and 5000 stretching cycles), and excellent response toward bending.

## 1. Introduction

With the rapid development of intelligent electronics, wearable electronic devices have sparked significant interest in academia and industry due to their extensive range of applications [1,2,3]. As a crucial component of wearable electronic devices, flexible strain sensors possess considerable potential for applications in intelligent robotics, electronic dermis, medical monitoring, and other fields. In particular, resistive strain sensors have attracted extensive research interest due to their simple readout systems and manufacturing processes [4,5,6,7]. To meet the requirements of practical applications, strain sensors need to have high sensitivity, a wide working range, and excellent mechanical properties. The brittleness and rigidity of traditional strain sensors made from metals or semiconductors limit their application in wearable devices. The tensile properties of flexible strain sensors can be significantly enhanced by incorporating conductive fillers into elastic polymer substrates [8].

Various wearable pressure sensors based on nanomaterials have been successfully developed. Nano-conductive fillers [9,10,11], such as metal nanoparticles [12,13,14,15], metal nanowires [16,17,18], conductive polymers [18,19,20,21], carbon nanotubes (CNTs) [22,23], and graphene [24,25], have been widely utilized in fabricating pressure sensors due to their exceptional electrical and mechanical properties. Metal nanowires are considered one of the most promising candidates among these nanofillers due to their exceptional electrical conductivity, flexibility, and ability for scalable synthesis. At the same time, flexible wearable films based on silver nanowires also have certain research applications in waterproof and antibacterial fields [22]. Different polymers such as polydimethylsiloxane (PDMS) [15], Ecoflex [16], polyurethane (TPU) [17], and silicone rubber are utilized in the fabrication of flexible substrates for strain sensors. For example, Kim et al. [18], incorporated AgNWs into a transparent polymer surface to enhance its adhesion and successfully developed a flexible sensor with exceptional mechanical properties. The results demonstrate the efficacy of employing microstructures to pattern the surface of the sensing layer in fabricating strain sensors, enabling enhanced sensitivity and an expanded working range. Lee et al. [19]. successfully controlled the embedding depth of surface CNT in a flexible sandwich sensor with good stability through precise adjustment of the pressure time of PDMS. Dai et al. [20]. developed a flexible strain sensor through the application of carbon nanotube ink onto an electrospun TPU fiber pad using spray technology. Subsequently, cracks were generated by employing a pre-stretching method. However, their sensing range is still very narrow, and it is difficult to meet the requirements of high sensitivity and wide flexibility. In addition, research is scarce on the study of the microstructure patterning of AgNWs to improve the performance of strain sensors. High-sensitivity strain sensors need to produce significant changes in the structure of the conductive network under stress stimulation. Simultaneously, the sensor needs to remain intact under stress to ensure effective electron transfer and accommodate a wide sensing range. Therefore, devising an effective strategy to ensure that the strain sensor exhibits both high sensitivity and a wide operating range remains a formidable challenge.

In this paper, a stretchable strain sensor composed of PDMS/AgNW/Pat-PDMS with a high gauge factor (GF) is designed by using a straightforward rod coating method to align AgNWs onto the surface of PDMS. The stretchable strain sensor is fabricated by embedding a microstructure of silver nanowires into the PDMS substrate. A stretchable conductive film, consisting of AgNW-PDMS, is prepared by employing a simple rod coating method to arrange the nanowires on the surface of PDMS. Depending on the angle of arrangement, the overlap area between the nanowires varies, allowing for different degrees of separation under a given strain. As the change in strain resistance primarily stems from nanowire separation and alterations in current path geometry, adjusting the angle θ of the nanowire enables precise regulation of strain. By altering the orientation of the Meyer rod coating, the microstructure of the AgNWs conductive film is engineered to enhance sensor sensitivity (achieving GFs of 15 at strain (ε) > 120%) and expand its operational range. We correlate the output signal with the relative change in resistance of the conductive film and validate its electrical and mechanical applicability through experimentation. To further validate the practical application of the sensor, the PDMS/AgNW/Pat-PDMS strain sensor is employed for detecting various motions. The sensor demonstrates significant potential in wearable electronics due to its outstanding sensitivity, cycle stability, and wide operating range.

## 2. Experimental Section

### 2.1. Reagents and Materials

The PDMS (SYLGARD 184, Goyang, Republic of Korea) was purchased from Dow Corning. Analytical pure silver nitrate (AgNO_3_, Aladdin Biochemical Technology Co., Ltd., Tianjin, China), analytical pure sodium chloride (NaCl•2H_2_O, Xilong Technology Co., Ltd., Guangzhou, China), and polyethylpyrrolidone (PVP, K60: Mw #60000, Xilong Technology Co., Ltd., Guangzhou, China) powders were supplied by Aladdin. Ethanol and ethylene glycol were purchased from Aladdin Chemical Co., Ltd. (Shanghai, China). Deionized water (18 MU cm) was obtained from a micro pure HIQ water purifying system.

### 2.2. Synthesis of AgNWs

AgNWs were synthesized in solution as follows: first, 2.0 g of polyvinylpyrrolidone (PVP) and 0.510 g of silver nitrate (AgNO_3_) were dissolved in 30 mL of ethylene glycol (EG) to form a transparent solution at room temperature. Then, AgNO_3_ solution was added to the PVP solution to induce the initial nucleation of silver seeds. Then, 0.030 mL of sodium chloride (NaCl, 0.023 mol/L) was added and stirred for 10 min. After mixing, the mixture was transferred to a 100 mL Teflon-sealed autoclave and heated at 160 °C for 2 h. After the reaction, the suspension was centrifuged at 6000 rpm (rpm: revolutions per minute) for 20 min, and the solid product was washed three times with both deionized water and ethanol to remove the remaining impurities such as EG and PVP in the supernatant.

### 2.3. Preparation of Patterned PDMS Film

The PDMS precursor was obtained by mixing the base and curing agent of Sylgard 184 (Dow Corning, Midland, MI, USA) at a ratio of 10:1, and the liquid mixture was degassed. The precursors were pre-polymerized at 60 °C for 30 min to form pre-cured PDMS (Pre-PDMS). The prepared Pre-PDMS film was cut into strips with the size of 5 cm × 3 cm. Then, the same area of a stainless steel mask was attached to the surface of Pre-PDMS and polymerized at 60 °C for 10 h. Then the composite film was immersed in hydrochloric acid to remove the stainless steel mask on the surface, and the PDMS film with a microscopic pattern (Pat-PDMS) was obtained.

### 2.4. Preparation of AgNW/Pat-PDMS Films

**Scheme 1:**
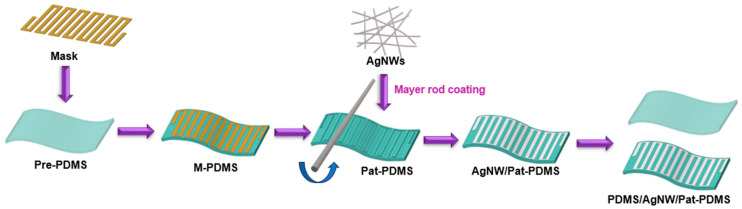
Schematic illustration for the flexible AgNW/Pat-PDMS film fabrication process.

The schematic diagram of the fabrication procedure for the AgNW/Pat-PDMS electrode is depicted in Scheme 1. In a typical synthesis procedure, the AgNWs solution with a concentration of 2 mg/mL was uniformly coated on the Pat-PDMS film with micro-grooves with a Meyer rod. Then, the AgNW/Pat-PDMS flexible electrode was obtained after drying in an environment with an oven temperature of 60 °C for 10 min. For comparison, the AgNW/PDMS electrode was also prepared using the same method, in which the flexible substrate was PDMS with a smooth surface.

### 2.5. Preparation of the PDMS/AgNW/Pat-PDMS Pressure Sensor

The prepared AgNW/Pat-PDMS film was placed on the pre-cured PDMS for sensor assembly of the sandwich structure, followed by a curing package. The specific steps are as follows: the Cu electrodes were attached to both sides of the prepared photographic AgNW/Pat-PDMS film using conductive adhesive. Subsequently, the pre-cured PDMS (prepolymer and crosslinker mixed at a 1:1 wt% ratio) was inverted and placed onto the surface of the AgNW/Pat-PDMS film and then heated at 60 °C for 10 h to cure encapsulation, resulting in the preparation of the PDMS/AgNW/Pat-PDMS composite film. Finally, the PDMS/AgNW/Pat-PDMS films were tested as strain sensors.

### 2.6. Characterization

The morphology of the materials was characterized using a scanning electron microscope (SEM), Hitachi SEM Su8010 (Tokyo, Japan), and the microstructure of the films was investigated. The crystalline phase and lattice parameter of the material were characterized by using an X-ray diffraction spectrometer (XRD, D8 Bruker, Billerica, MA, USA), where the XRD peaks ranged from 10° to 80° with a step size of 0.02°. The chemical composition of the electrodes was determined using XPS spectroscopy (XPS Thermo Fisher, E. Grinstead, UK). The resistance of the strain sensor was measured using the four-probe method. The four-probe method is the most commonly employed technique for measuring the resistance of diffused layers in thin films. In this method, four equidistant metal probes make contact with the silicon surface, with the outer two probes carrying a DC I and the voltage drop V across the middle two probes being measured using a potentiometer. The stress–strain curve and electromechanical properties of the strain sensor were determined using a general material testing machine (HY-0350, Shanghai Hengyi Precision Instrument Co., Ltd., Shanghai, China) and a digital source meter (2400, Keithley, Tektronix, Cleveland, OH, USA).

## 3. Results and Discussion

### 3.1. Structure and Morphology

Scheme 1 involves the fabrication of a surface-patterned AgNW/PDMS film diagram. A patterned groove structure (Pat-PDMS) was created using a mask on the surface of pre-cured PDMS (Pre-PDMS). The groove structure facilitates the enhancement of adhesion between the PDMS substrate and AgNWs, thereby improving the mechanical properties of the flexible substrate. Subsequently, a Meyer rod was employed to control the coating direction of the AgNWs solution, resulting in a microstructure of AgNWs with directional arrangement. To investigate the impact of pattern shape on sensor sensitivity, we developed arrangement patterns at various angles (0°, 60°, and 90°) between AgNWs to fabricate AgNW/Pat-PDMS films. When the film is stretched, the degree of separation and sliding between conductive materials can be adjusted by control, thereby improving the accuracy of conductivity adjustment in the film [21]. The sensitivity of strain sensors utilizing AgNW/Pat-PDMS films can thus be enhanced.

**Figure 1:**
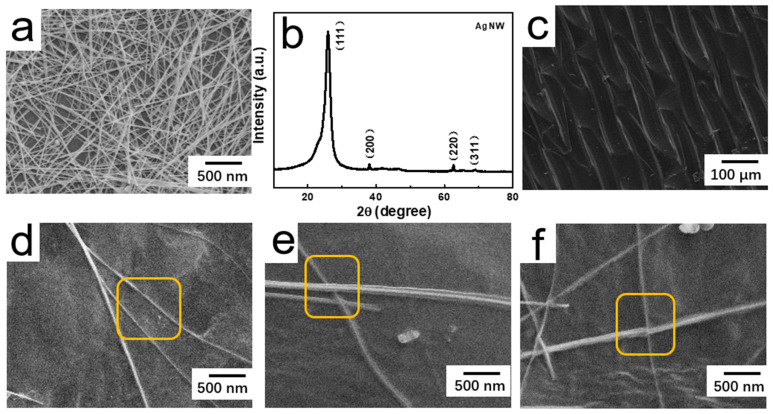
SEM images and XRD: AgNWs (**a**,**b**); SEM images: Pat-PDMS (**c**); AgNW/Pat-PDMS ((**d**) 0°; (**e**) 60°; (**f**) 90°); yellow frames: Silver nanowires distributed at different angles.

To understand the morphology of surface-patterned PDMS and AgNWs distribution, the AgNW/PDMS films were characterized using the SEM. The morphology of the synthesized AgNWs is depicted in Figure 1a, revealing a smooth surface and a length-diameter ratio of approximately 1000. XRD characterization was conducted to ascertain the synthesized AgNWs solution’s composition further, as illustrated in Figure 1b. As can be seen from Figure 1b, the diffraction patterns of the films are indexed to face-centered cubic Ag (JPDCS No. 04-0783) [9]. The obvious four peaks at 38.11°, 44.30°, 64.44°, and 77.40° corresponded to (111), (200), (220), and (311) Bragg reflections of Ag.

The surface of PDMS was patterned with grooves using a stainless steel mask. The specific experimental method can be found in our previous research report. As depicted in Figure 1c, the average diameter of the groove is ~50 μm. The groove structure on the PDMS surface enhances the adhesion of AgNWs and prevents their shedding during the stretching process. The AgNWs were then applied to the grooves on the PDMS surface using a rod coating method. By adjusting the coating direction of the Meyer rods, a conductive network composed of AgNWs arranged at different angles (0°, 60°, and 90°) was prepared, as shown in Figure 1d–f.

The atomic species and bonding characteristics of AgNW/Pat-PDMS were determined through XPS analysis, which detected typical Ag, C, O, and Si elements in the binding energy range of 0–550 eV (Figure S1a). Additionally, high-resolution analysis of Ag3d was conducted to further determine the valence states of elements in AgNW/Pat-PDMS. Figure S1b shows two peaks at 368.2 and 374.2 eV, corresponding to the Ag 3d5/2 and Ag 3d2/3 characteristics, respectively (Figure S1b) [15]. These results indicate the successful preparation of AgNW/Pat-PDMS films.

### 3.2. The Optoelectrical Properties of Electrodes

**Figure 2:**
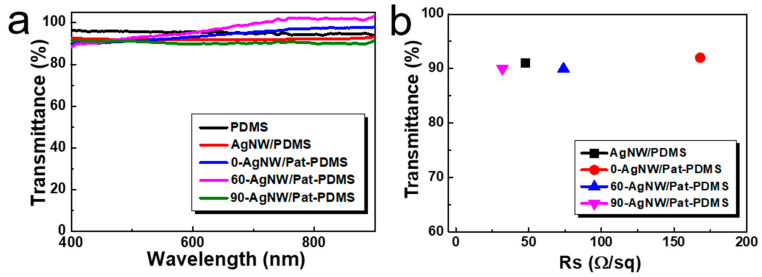
Transmittance spectra (**a**); transmittance and sheet resistance (**b**) of AgNW/PDMS, 0-AgNW/Pat-PDMS, 60-AgNW/Pat-PDMS, and 90-AgNW/Pat-PDMS films.

The transmittance and conductivity of the prepared AgNW/Pat-PDMS films were assessed. To investigate the impact of nanowire orientation on conductivity, the dosage of AgNWs was initially optimized, with the results presented in Figure S2. AgNWs with a concentration of 2 mg/mL and a volume of 0.6 mL were chosen for the coating of the film. The transmittance of PDMS, AgNW/PDMS, 0-AgNW/Pat-PDMS, 60-AgNW/Pat-PDMS, and 90-AgNW/Pat-PDMS films were compared, as illustrated in Figure 2a. The transmittance of different films at 550 nm wavelength is 95%, 91%, 92%, 90%, and 90%, respectively, which indicates that the effect of nanowire structures arranged at different angles on the transmittance of the films is negligible. The initial resistance measurement results of different films are shown in Figure 2b. The initial resistances of AgNW/PDMS, 0-AgNW/Pat-PDMS, 60-AgNW/Pat-PDMS, and 90-AgNW/Pat-PDMS films are 48 Ω/sq, 168 Ω/sq, 74 Ω/sq, and 32 Ω/sq, respectively. The reason may be that the nanowire forms a conductive network in different ways, and the contact area formed is different, resulting in differences in resistance.

**Figure 3:**
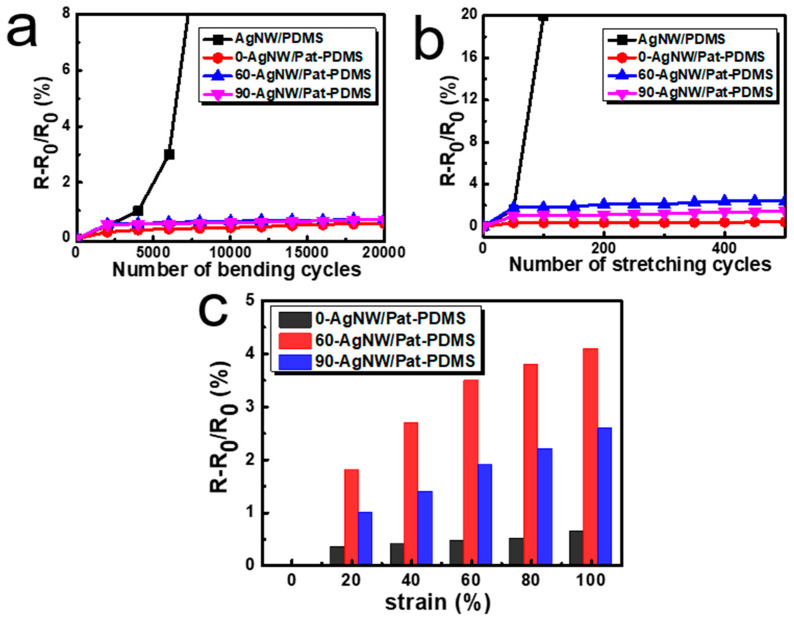
Relative resistance changes under bending tests with 20,000 bending cycles (**a**); stretching tests with 500 cycles (**b**); different tensile strains from 20% to 100% (**c**) of AgNW/PDMS, 0-AgNW/Pat-PDMS, 60-AgNW/Pat-PDMS, and 90-AgNW/Pat-PDMS films.

Depending on the orientation angle, the overlap area between nanowires varies, resulting in different levels of separation under a given strain. Due to the separation of the nanowire and the change in current path geometry, the variation in strain resistance of the sensor can be primarily attributed to these factors. Therefore, precision in strain regulation can be adjusted by altering the angle θ (0°, 60°, 90°) of the nanowire. In this study, we initially enhanced the mechanical stability of the film by implementing patterned groove designs on the surface of pre-cured PDMS. The mechanical durability of the film is illustrated in Figure 3a. Specifically, Figure 3a depicts the changes in resistivity of AgNW/Pat-PDMS films measured under prolonged bending conditions. The initial square resistance change rates of AgNW/PDMS, 0-AgNW/Pat-PDMS, 60-AgNW/Pat-PDMS, and 90-AgNW/Pat-PDMS films were 0.46, 0.23, 0.51, and 0.49, respectively, under the bending condition with a curvature radius of 2 mm. After 20,000 repeated bending tests, the final square resistance change rates were 183, 0.53, 0.69, and 0.58, respectively. The three films exhibited consistent rates of change with minimal variation between them. Meanwhile, it was noted that the square resistance of AgNW/PDMS films shows a significant disparity before and after the rate of change, indicating the detachment of nanowires leading to the disruption of the conductive network.

The resistivity changes of AgNW/Pat-PDMS films under cyclic tensile conditions are illustrated in Figure 3b. The film was elongated to 20% and then returned to its initial length. The film was stretched and restored for 500 cycles, and the results are presented in Figure 3b. The electrical conductivity of AgNW/PDMS films diminishes after undergoing two rounds of stretching. The initial square resistance change rates for 0-AgNW/Pat-PDMS, 60-AgNW/Pat-PDMS, and 90-AgNW/Pat-PDMS films are 0.35, 1.8, and 1.1, respectively. Following 500 tensile tests, the final square resistance change rates were measured at 0.65, 4.1, and 2.6, respectively. Among these results, the film with a composition of 60-AgNW/Pat-PDMS exhibited the most significant change in square resistance. The 0-AgNW/Pat-PDMS, 60-AgNW/Pat-PDMS, and 90-AgNW/Pat-PDMS films were subsequently subjected to varying degrees of stretching, and the resulting changes in square resistance were measured. With the increase in tensile strain (stretching from 20% to 100%), the overall resistance of the three films was found to increase, albeit with varying degrees of change. The 0-AgNW/Pat-PDMS and 90-AgNW/Pat-PDMS films exhibited a relatively gradual increase, while the 60-AgNW/Pat-PDMS film showed a more significant change. From Table 1, the resistivity changes of different films under long-term bending, repeated stretching, and different angles can be more clearly compared.

The reason for the decrease in the resistance of the film during bending and stretching may be that the separation occurs at the cross-contact of the nanowire, resulting in changes in the conductive network and thus in the conductivity. The varying rates of resistance change in different films during bending and stretching may be attributed to the differing degrees of contact point separation and the conductivity decline of nanowires arranged at different angles.

### 3.3. Sensor Application Performance

**Figure 4:**
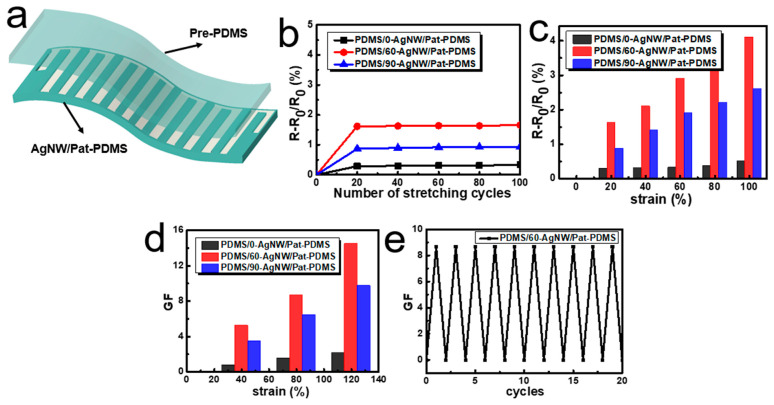
Sensor structure diagram (**a**); relative resistance changes under stretching tests with 100 cycles (**b**); different tensile strains from 20% to 100% (**c**); GF under different tensile strains from 40% to 120% (**d**) of 0-AgNW/Pat-PDMS, 60-AgNW/Pat-PDMS, and 90-AgNW/Pat-PDMS films; GF with tensile strains 40% of 60-AgNW/Pat-PDMS (**e**).

The wearable piezoresistive strain sensor is suitable for measuring human movement due to its excellent flexibility, high sensitivity, and wide strain range. A sandwich structure in the form of PDMS/AgNW/Pat-PDMS was designed to achieve a flexible and stretchable piezoresistive strain sensor, as illustrated in Figure 4a. Pre-cured PDMS was utilized as the initial substrate to offer mechanical support for the sensor and to provide the necessary flexibility for the compression resistance of the AgNW/Pat-PDMS thin layer. The use of pre-cured PDMS can improve adhesion to AgNW/Pat-PDMS films. The sensitivity of a strain sensor is typically characterized by the relative change in resistance (R − R_0_)/R_0_, where R_0_ and R represent the initial and final resistances under strain, respectively. The sensor exhibits stable mechanical properties, as evidenced in Figure 4b, where the relative resistance changes at different angles are 0.29, 1.62, and 0.87 after 100 repeated stretches within the range of 20%. Simultaneously, the sensor’s relative resistance uniformly varies across the 20% to 100% stretch range, as depicted in Figure 4c, primarily due to the contribution of one-dimensional nanowires to the continuity of the conductive path.

The sensitivity of the stretchable strain sensor is generally assessed by the gauge factor (GF), which is defined as the ratio of the relative change in resistance to the mechanical strain (GF = (R − R_0_)/R_0_)/ε, where ε represents the applied mechanical strain [21]. The gauge factor value of the strain sensor varies under different levels of mechanical strain (Figure 4d). At a small strain of ~40%, the gauge factor (GF) of the three sensors is approximately 0.75, 5.25, and 3.5, respectively. As the strain increases to ~80%, the GF increases to around 1.52, 8.67, and 6.45 for each sensor. Upon reaching a tensile strain of ~120%, the GF further rises to approximately 2.12, 14.52, and 9.76 for each sensor. These findings demonstrate that the PDMS/60-AgNW/Pat-PDMS sensor exhibits heightened sensitivity in monitoring strains ranging from 0 to 120%. The reason may be that the PDMS/0-AgNW/Pat-PDMS sensor has a small nanowire overlap region, and the large deformation separates the adjacent nanowire, causing the conductive path of the sensor to disconnect. The PDMS/90-AgNW/Pat-PDMS sensor has a large overlap area, and a small amount of separation occurs between the nanowires during stretching. The PDMS/60-AgNW/Pat-PDMS sensor shows high adjustment accuracy. The mechanical stability of the PDMS/60-AgNW/Pat-PDMS sensor was also investigated. The sensor demonstrated stable cyclic performance at strains of 40% after 20 cycles (Figure 4e). Therefore, the proposed directionally aligned PDMS/60-AgNW/Pat-PDMS sensor demonstrates the capability to accurately detect subtle pressure movements, suggesting its potential for a wide range of applications. The PDMS/60-AgNW/Pat-PDMS sensor’s performance is comparatively favorable compared to previously reported works (refer to Support Information Table S1) [26,27,28,29,30,31].

## 4. Conclusions

In conclusion, a stretchable sensor was fabricated using a micro-directional arrangement of silver nanowires in an AgNW/Pat-PDMS thin film through a simple Meyer rod coating method. The separation and deformation degree of the nanowire under a given strain can be further controlled by adjusting the orientation angle of the nanowire, thereby enhancing the precision of sensor resistance change. The PDMS/60-AgNW/Pat-PDMS strain sensor exhibits a high gauge factor of up to 14.52 and an operating strain range of up to 120%. Furthermore, the strain sensor demonstrates excellent cyclic stability, enduring 2000 bending cycles and 500 stretching cycles without significant performance degradation. The strain sensor is constructed by embedding silver nanowire microstructures into PDMS substrates, resulting in a stretchable and flexible design. By adjusting the arrangement angles of the nanowires, varying degrees of separation can be achieved under different strains, thereby enhancing the accuracy and precision of the sensor. Therefore, the PDMS/AgNW/Pat-PDMS strain sensor, prepared through the directional arrangement of silver nanowires, exhibits significant potential for application in future wearable electronic devices.

## Figures and Tables

**Table 1:** Comparison of the resistivity changes of different films under long-term bending, repeated stretching, and different strains.

Samples	20,000 Bending Cycles (%)	500 Stretching Cycles (%)	20~100% Tensile Strain (%)
AgNW/PDMS	183	-	-
0-AgNW/Pat-PDMS	0.53	0.65	0.49~0.71
60-AgNW/Pat-PDMS	0.69	4.1	1.81~4.23
90-AgNW/Pat-PDMS	0.58	2.6	0.98~2.45

## Data Availability

The original contributions presented in the study are included in the article/Supplementary Material, further inquiries can be directed to the corresponding authors.

## Supplementary Materials

The following supporting information can be downloaded at: https://www.mdpi.com/article/10.3390/ma17164059/s1. Figure S1: XPS spectra of AgNW/Pat-PDMS (a) and Ag 3d (b); Figure S2: Sheet resistance of AgNW/PDMS film under different amounts of AgNWs; Table S1: Comparison of resistivity changes of different films under long-term bending, repeated stretching of various reported works in comparison with this work.
