# Supplementary material for: Flexible Transparent Films of Oriented Silver Nanowires for a Stretchable Strain Sensor
Source: Materials (Basel). 2024 Aug 15;17(16):4059. doi: 10.3390/ma17164059 (PMC11355971; doi:10.3390/ma17164059)
Supplement: Supplementary file 1 [file materials-17-04059-s001.zip › materials-3096929-supplementary.pdf]

materials

Supplementary Materials

Flexible Transparent Films of Oriented Silver Nanowires for a Stretchable Strain Sensor

Xiaoguang Wang [1], Chengjun Song [1], Yangyang Wang [1], Shaoxuan Feng [1], Dong Xu [1], Tingting Hao [1,*] and Hongbo Xu [2,*]

[1] China Electronic Technology Group Corp 49th Research Institute, Harbin 150001, China
[2] School of Chemistry and Chemical Engineering, Harbin Institute of Technology, Harbin 150001, China
* Correspondence: htt12030810@163.com (T.H.); iamxhb@hit.edu.cn (H.X.)

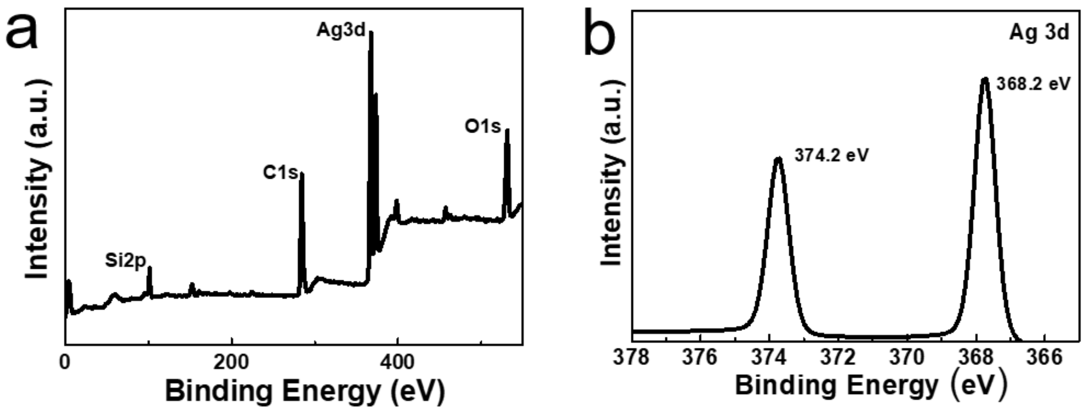

Figure S1. XPS spectra of AgNW/Pat-PDMS (a) and Ag 3d (b).

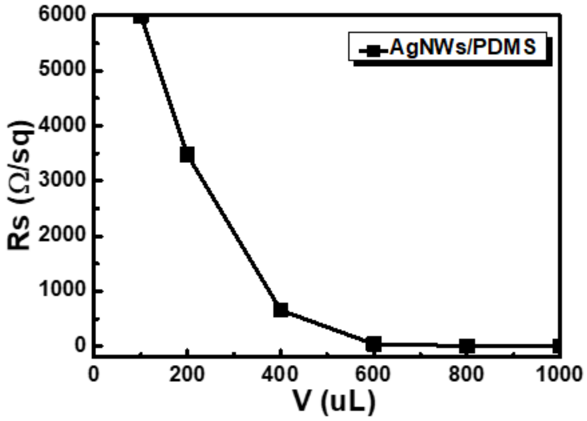

Figure S2. Sheet resistance of AgNW/PDMS film under different amounts of AgNWs.

Table S1. Comparison of resistivity changes of different films under long-term bending, repeated stretching of various reported works in comparison with this work.

Samples	Bending Cycles	Tensile Strain	GF	Ref.
CNT/TPU	10000		45	[26]
CNT/PDMS	1000	20~100%	36	[27]
TPU/MWCNT	250	0~100%	-	[28]
NP/PDMS	5000	0~70%	-	[29]
AgNW/CNT/PDMS	5000	0~70%	-	[30]
CNT/Ecoflex	1000	0~500%	64	[31]
60-AgNW/Pat-PDMS	20000	20~100%	97	Our work
